# Supplementary figures and images for: Glucose-lactose mixture feeds in industry-like conditions: a gene regulatory network analysis on the hyperproducing Trichoderma reesei strain Rut-C30
Source: BMC Genomics. 2020 Dec 10;21:885. doi: 10.1186/s12864-020-07281-8 (PMC7731781; doi:10.1186/s12864-020-07281-8)

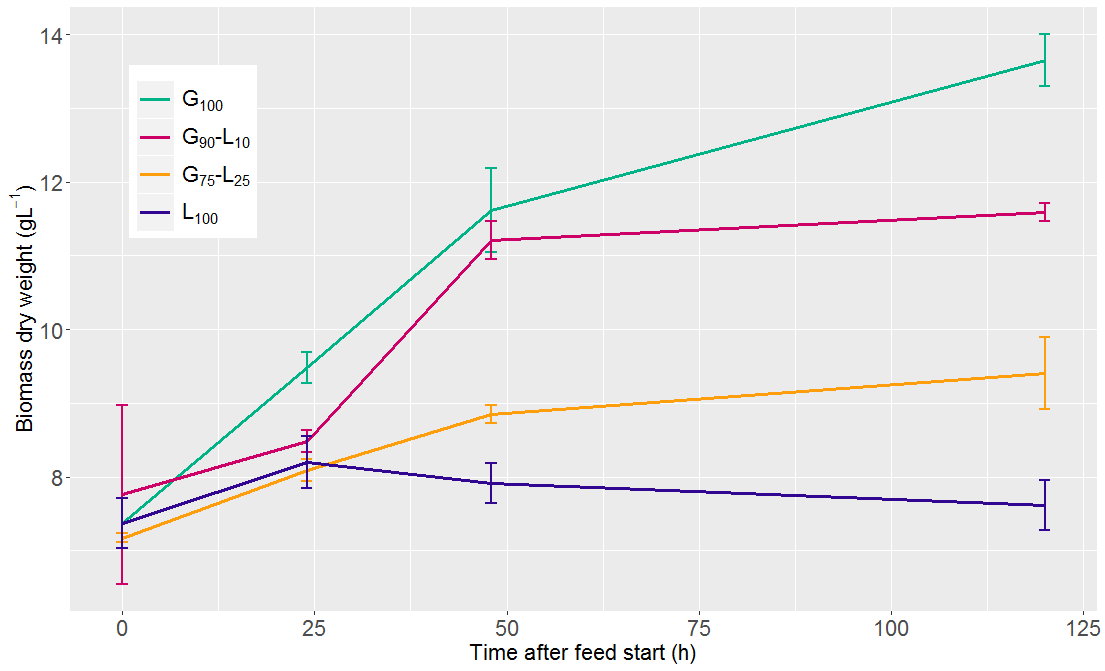

Supplement: Supplementary file 1 — Additional file 1 Study of the biomass concentration during the fed-batch. This PNG file contains experimental results regarding the study of the Rut-C30 biomass concentration at 0 h, 24 h, 48 h and 120 h during the fed-batch on G100, G75-L25, G90-L10 and L100. [file 12864_2020_7281_MOESM1_ESM.png]

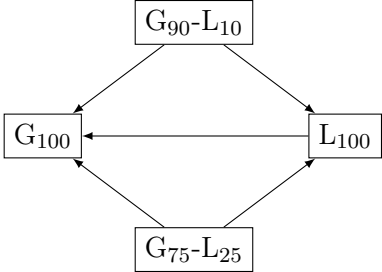

Supplement: Supplementary file 2 — Additional file 2 Circuit design. This PDF file contains an illustration of the methodology used to perform the differential analysis. [file 12864_2020_7281_MOESM2_ESM.pdf]
